# Experience and training needs of nurses in military hospital on emergency rescue at high altitude: a qualitative meta-synthesis

**DOI:** 10.1186/s12912-024-02029-1

**Published:** 2024-06-03

**Authors:** Ruixuan Zhao, Shijie Fang, Dongwen Li, Cheng Zhang

**Affiliations:** 1 North Sichuan Medical University, Nanchong, Sichuan 637000 China; 2 General Hospital of Western Theater Command, Chengdu, Sichuan 610083 China; 3 Chengdu Medical College, Chengdu, Sichuan 610500 China

**Keywords:** Military nurse, High altitude, War wound, First aid, Experience, Training needs, Meta-synthesis

## Abstract

**Background:**

Nurses play an important role in the treatment of war wounds on the plateau, and they face multiple challenges and a variety of needs in their caregiving process. This study aimed to systematically integrate and evaluate qualitative research data to understand the altitude emergency rescue experience and training needs of nurses in military hospitals and provide them with targeted assistance.

**Methods:**

We critically assessed the study using the Joanna Briggs Institute Critical Assessment Checklist for Qualitative Research. Extraction, summarization and meta-synthesis of qualitative data. Cochrane Library, PubMed, Embase, FMRS, CINAHL, PsycINFO, Chinese National Knowledge Infrastructure (CNKI), Wanfang Database (CECDB), VIP Database, and China Biomedical Database (CBM) were searched for relevant studies published from the establishment of the database to May 2023. Additionally, we conducted a manual search of the references of the identified studies. Registered on the PROSPERO database (CRD42024537104).

**Results:**

A total of 17 studies, including 428 participants, were included, and 139 research results were extracted, summarized into 10 new categories, and formed 3 meta-themes. Meta-theme 1: mental state of military nurses during deployment. Meta-theme 2: the experience of military nurses during deployment. Meta-theme 3: training needs for emergency care.

**Conclusions:**

Emergency rescue of high-altitude war injuries is a challenging process. Leaders should pay full attention to the feelings and needs of military nurses during the first aid process and provide them with appropriate support.

**Supplementary Information:**

The online version contains supplementary material available at 10.1186/s12912-024-02029-1.

## Introduction

The plateau area has the characteristics of high altitude, cold all the year round, many ice peaks and snow mountains, and hypoxia [1]. These characteristics pose major obstacles to both military operations and non-military operations and at the same time, due to the complex terrain and inconvenient transportation, the detection, handling, treatment, and evacuation of the wounded become very difficult. These special natural environments put forward higher requirements for medical rescue [2, 3]. As an important part of military or non-military missions, military nurses play an important role in emergency rescue [4]. There has been a long history of military nurses engaging in war, military operations and humanitarian missions, they are required to provide not only routine health care during peacetime, but also medical services during conflict or humanitarian assistance in response to disasters, public emergencies and epidemics [5, 6]. The rescue process is arduous, and nurses may face great challenges. When they are at high altitude environment, they are prone to hypoxia, frostbite, sunburn, fall, blindness, etc., and may be accompanied by high altitude pulmonary edema and high-altitude coma. In war and non-war military operations, military nurses are required to care for a variety of trauma patients, including burns, traumatic amputations, shock, bleeding, penetrating injuries, spinal cord injuries, head injuries, crush injuries, radiation injuries, chemical injuries, infectious diseases, and more. This has higher requirements for the physical, psychological and professional knowledge of military nurses [4, 7, 8].

To provide better care for the wounded and respond to various emergency situations, military nurses must continuously improve their competence. In addition, according to the literature [4, 6, 9], the demand of military nurses for emergency rescue training is gradually increasing, with nurses with deployment experience reporting limited first aid proficiency and a lack of practical training, and related qualitative studies are also increasing, but a single qualitative research result is difficult to fully and accurately reflect the needs of military nurses. Therefore, this study uses a meta-synthesis approach to analyze and summarize such studies the to understand experience and training needs of nurses in military hospitals with altitude war injury emergency rescue, to provide reference for formulating altitude emergency rescue training strategies, and better meet their needs and provide them with appropriate support.

## Methods

### Study design

The Joanna Briggs Institute(JBI)methodology for systematic reviews of qualitative evidence [10] guided this systematic review and qualitative meta-synthesis. We used the PROSPERO to identify published or ongoing research relevant to the topic and registered for this review(CRD42024537104). In addition, we report our findings by the Enhancing Transparency in Reporting the Synthesis of Qualitative Research (ENTREQ) Statement [11].

### Search strategy

We performed systematic searches in Cochrane Library, PubMed, Embase, FMRS, CINAHL, PsycINFO, Chinese National Knowledge Infrastructure (CNKI), Wanfang Database (CECDB), VIP Database, and China Biomedical Database (CBM). The retrieval time limit was from the establishment of the database to May 2023. The following search terms were used in different combinations: plateau, qualitative study, Emergency rescue, train, Military nurses, education, disaster, public health emergency, rescue, army, War readiness, war. Additionally, we conducted a manual search of the references to the identified studies to find additional eligible articles.

### Inclusion and exclusion criteria

Articles that satisfied the following criteria were included in the qualitative synthesis: 1)study population(P): military nurses; 2)phenomenon of interest(I): highland or mountain emergency rescue or emergency rescue experiences, experiences and training needs; 3)context(Co): military nurse emergency rescue process or training process; 4)type of study: qualitative research, including phenomenological, descriptive qualitative research, rooted theory, ethnography, etc.

The exclusion criteria were as follows: 1)duplicate literature, literature with unavailable full text or incomplete data, literature with substandard quality (The JBI qualitative research critical assessment is graded C); 2)literature not in English; 3) secondary research.

### Article filtering and quality assessment

Literature screening was done independently by 2 researchers following strict inclusion and exclusion criteria, and they independently assessed the quality of the included literature using the JBI Manual for Systematic reviews of qualitative evidence [10]. The guideline has 10 evaluation items, each items uses “yes”, “no”, and “not provided” as evaluation indicators. In this study, literature quality is divided into A, B and C. A represents that the literature meets all the above evaluation indicators, B represents that the literature partially meets, and C represents that it does not meet all the above evaluation indicators. During the article selection and quality evaluation process, disagreements were settled with discussion or with a third author’s assistance.

### Data extraction

Data management was enabled by the reference management program Endnote 20. Data extraction consists of two researchers reading the content contained in the study independently to extract relevant and useful information, cross-reviewed, and when any disagreement was discussion to resolve it with a third experienced researcher. The relevant content of each study was extracted using a standardized data extraction tool from the Joanna Briggs Institute Qualitative Assessment and Review (JBI-QARI), the JBI-QARI qualitative criteria are: (1) unequivocal (U)—refers to findings that are a matter of fact, beyond a reasonable doubt; (2) credible (C)—refers to findings that are plausible interpretations of the primary data within the theoretical framework; (3) unsupported (Un)—relates to findings that are unsupported by the data [12]. The researchers extracted data according to the above criteria. Data extraction included author, country, objective, study population, research Methodology, and main results.

### Data synthesis

This data extraction was carried out and checked independently by 2 researchers, and when disagreements were encountered, a third researcher was asked and consensus was reached on the results. We used Thomas and Hardens’ three stage thematic synthesis approach [13]: (1) coding the text; (2) developing descriptive themes; (3) generating analytical themes. First, two researchers independently coded the results based on text content and meaning; then, researchers looked for similarities and differences between the textual data, and classify the meaning of the original dataset; finally, the categories were evaluated repeatedly to identify similarities and obtain synthesized results.

## Results

### Study characteristics

**Fig. 1:**
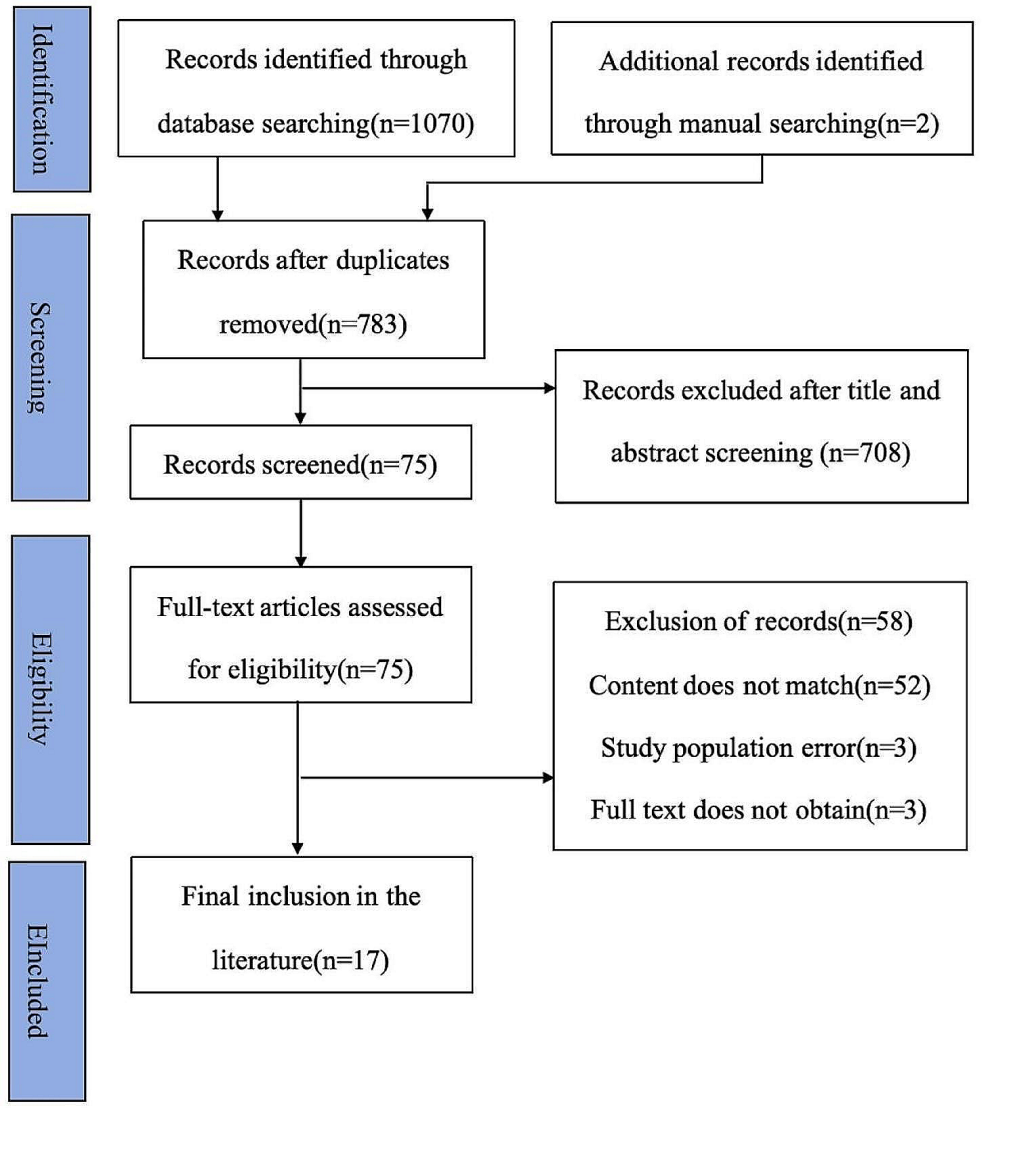
PRISMA flowchart and literature selection results

A total of 1070 articles were searched, we found two additional articles by checking the references of articles, and the exclusion of duplicate publications yielded 783 articles. After reading the titles and abstracts, 708 articles were excluded. After reading the remaining 75 articles 58 articles were excluded, including 52 articles with content mismatches, 3 articles studied population errors and full text information could not be obtained for 3 articles, Finally, 17 studies [7, 9, 14–28] were identified for inclusion in this analysis. The results of the search are shown in the PRISMA flowchart in Fig. 1. The 17 included studies were published between 2005 and 2023, of which 16 were qualitative studies [7, 9, 14–26, 28] and one were mixed-methods studies [27]. A total of 428 participants, involved 6 countries, including China (2 study [7, 27]), USA (6 studies [9, 15, 17, 20–22]), Sweden (2 studies [14]), Iran (3studies [19, 24, 25]), Israel (1 study [28]), Korean (2 studies [23, 26]), and British (1 study [18]). The characteristics of the included literature are shown in Table 1.

### Quality assessment of studies

The included studies were evaluated separately by two trained researchers using the JBI Qualitative Research quality Evaluation criteria, who then participated in the discussion together. When disagreements arose, the help of a third researcher was sought and the final results were unanimously approved by the researchers. All literature included in this study was either A or B grade, which three studies were quality rating of A and 13 studies with a B. Table 2 presents the results of the critical appraisal of the 17 studies.

**Table 1:** Characteristics of the included studies

Authors, year, country	Objective	Data collection method and techniques	Population	Main results
Lindblad et al [14]. (2005), Sweden	Explore how nurses perceive battlefield emergency care.	Descriptive study/in-depth interview method.	11 male registered nurses who all had been trained as company nurses in the Armed Force.	3 results: Unpredictable and invisible; contextual leadership; assimilation acts.
Ekfeldt et al [16]. (2015). Sweden	describe military nurses’ experiences of preparations for a mission, including factors that contribute to their being meaningful.	Qualitative study/semistructured interviews	8 nurses were interviewed in this study.	2 categories: The first category illustrates how the nurses recognize challenges that they have to prepare for. The second concerns making informed choices to be adequately prepared.
Ma et al [27]. (2023), China	To obtain a deeper understanding of the past experiences and future expectations of Continuing professional education among nurses in Chinese military hospitals.	Qualitative study/Semi-structured interviews.	20 nurses were interviewed in this study.	5 results: they wanted to learn about in the future were military missions; military training content; military medicine training content; training methods; professional development paths.
Xing et al [7]. (2022), China	To explore the status of battlefield rescue capabilities of non-active military nursing personnel in the northwest plateau theater, and provide a basis for nursing training to standardize, supplement, and promote the effectiveness of the rescue effectiveness of the Northwest Plateau battlefield.	Qualitative study/semi-structured interviews.	18 army civilian nurses in this study.	5 themes: were army civilian nurses’ strong sense of pride and mission, high pressure to treat battle wounds on the field, psychological pressure and adequate reserve of expertise in war wound treatment, lack of field survival ability on the plateau battlefield, and lack of religious belief and common language communication with ethnic minorities.
Segev [28]. (2023). Israel	explore the lessons of the experiences of civilian nurses deployed to Israeli battlefields in three wars between 1967 and 1982.	Qualitative study/semi-structured interviews, in-depth interviews.	22 nurses were interviewed in this study.	3 results: Field Service Challenges, Coping with Challenges, and Nurses’ Need for Recognition.
Tow et al [21]. (2016). USA	Described the lived experience of U.S. warrior nurses who served as advisors to host nation officials in Afghanistan.	Phenomenological research/in-depth interviews.	10 nurses were interviewed in this study.	2 themes:3 composite textural descriptions: challenging role, challenging place, and changed.8 composite Structural description be: careful, be powerful, be courageous, be resourceful, be impeccable, be malleable, be caring, and be resilient.
Scannell-Desch [15]. (2005). USA	The aim of this paper is to describe guidance for nurses today from the lessons learned by nurses who served in the Vietnam War.	Hermeneutic phenomenological research/semistructured interviews.	24 nurses were interviewed in this study.	7 results: advice about journaling, training, caring for yourself, use of support systems, talking about your experiences, understanding the mission, and lack of preparation for war.
De Jong et al [9]. (2010) USA	This study was to document personal accounts of experiential learning concerning medical and nursing care rendered during combat operations in order to describe, evaluate, extend, and disseminate new knowledge for further development.	Ethnographic approach/focus group interviews	107 Air Force, Army, and Navy nurses were interviewed.	8 results: organizing for mass casualties, uncertainty about incoming casualties, developing systems to track patients, resource utilization, ripple effects of a mass casualty event, enlarging the scope of nursing practice, operating medical facilities under attack, and nurse emotions related to mass casualties.
Rivers et al [22]. (2017) USA	Explore military nurses’ perceptions of similarities, differences, and resulting issues of military deployments.	Phenomenological research/semistructured interviews	65 nurses were interviewed in this study.	7 results: Similarities: We Have Suffered, Support Really Matters, The Chaos is Real, and I’m a Different Person Now ; Differences: We Didn’t Know, The Structure is Missing, Disasters and War Are Not Equal.
Rivers [20]. (2016). USA	illuminate the nurses’ experiences in responding to disasters.	Phenomenological research/in-depth interviews.	23 nurses were interviewed in this study.	6 results: Nature of War Versus Nature of Disaster; Known Versus Unknown; Structured Versus Chaos; Prepared Versus Making Do; Being Strong Versus Expressing Emotion; Existential Growth.
Elliott [17]. (2015). USA	Describe the military nurses’ post-deployment experiences and their meaning.	Descriptive research/semistructured interviews	10 nurses were interviewed in this study.	5 results: learning to manage changes in the environment, facing the reality of multiple losses, feeling like it’s all so trivial now, figuring out where I ‘fit’ in all the chaos, and working through the guilt to move forward.
Vafadar et al [24]. (2021). Iran	Explore the experiences of the military nurses of participating in an interprofessional education program in crisis management domain.	Qualitative study/focus group interviews.	28 military nurses were interviewed in this study.	4 results: professional mutual recognition, shared mental models, valuing joint responsibility and collaboration, perceived self-worth as a member of an interprofessional team.
Rahimaghaee et al [19]. (2016). Iran	explains nurses’experiences and views on the care of injured soldiers during the Iraq–Iran war (1980–1988).	Descriptive research/semistructured interviews	14 nurses were interviewed in this study.	2 themes (Care in the war, a different culture and concept and Care achievements during the war) and 6 subthemes (Unusual working conditions, Different work spirit, A real but informal classroom, Professional self-achievements, Outcomes for the professional community, The changed self).
Varpio et al [25]. (2021). Iran	Lessons learned from military care providers providing care to patients in large-scale emergencies.	Grounded Theory approach/semistructured interviews.	30 participants were interviewed.	2 results: own your purposes and responsibilities (through mission focus and ethical bearing) and get it done, safely (via situational awareness, adaptability, and leadership with followership.
Han [23]. (2019) Korean	Exploring the experience of Korean nursing officers’ participation.	Hermeneutic phenomenological research/semistructured interviews.	14 nurses were interviewed in this study.	7 results: Enduring confusion, being devoted to duty, establishing deep comradeship, Realizing the dark side of war, being discriminated against as female, Achieving and being rewarded, and growing as leaders.
Kwon et al [26]. (2022). Korean	explored the experiences of new nurses with less than one year of clinical experience in caring for COVID-19 patients in a military hospital.	Phenomenological research/in-depth interviews.	6 nurses were interviewed in this study.	12 results: Fear of a new circumstance; Communication difficulties with isolated patients; Nervous about caring for unfamiliar, critically ill patients; Physically exhausted; Psychological withdrawal; Studying hard to provide skilled nursing; Searching for own know-how; Showing comradeship and encouraging each other; Gaining confidence; Striving for patient-centered care; Thinking critically; Feeling proud as a military nursing officer.
Finnegan et al [18]. (2015). British	To provide an analysis of the impact and effectiveness of the pre-deployment educational preparation and clinical placements provided for military nurses.	Grounded Theory/semistructured interviews	18 nurses were interviewed in this study.	4 results: Military Nursing Care; Military Nursing Education; Clinical Placements; Unique Hospital Environment.

**Table 2:** Results of quality assessment based on JBI critical appraisal checklist for qualitative studies

Literature	①	②	③	④	⑤	⑥	⑦	⑧	⑨	⑩	Quality
Lindblad et al [14]. (2005)	Y	Y	Y	NP	Y	Y	N	Y	Y	Y	B
Ma et al [27]. (2023)	Y	Y	Y	Y	Y	N	NP	Y	Y	Y	B
Xing et al [7] (2020)	Y	Y	Y	Y	Y	N	N	Y	Y	Y	B
Varpio et al [25] .(2021).	Y	Y	Y	Y	Y	Y	Y	Y	Y	Y	A
Segev [28].(2023)	Y	Y	Y	Y	Y	N	N	Y	Y	Y	B
Scannell-Desch [15].(2005)	Y	Y	Y	Y	Y	N	N	Y	Y	Y	B
Han [23]. (2019)	Y	Y	Y	NP	Y	N	NP	Y	Y	Y	B
Tow et al [21].(2016)	Y	Y	Y	Y	NP	N	N	Y	Y	Y	B
De Jong et al [9]. (2010)	Y	Y	Y	Y	Y	NP	N	Y	Y	Y	B
Rivers et al [22]. (2017)	Y	Y	Y	NP	Y	N	NP	Y	Y	Y	B
Vafadar et al [24] .(2021).	Y	Y	Y	Y	Y	N	Y	Y	Y	Y	B
Elliott [17]. (2015)	Y	Y	Y	Y	Y	N	NP	Y	Y	Y	B
Rahimaghaee et al [19]. (2016).	Y	Y	Y	NP	Y	N	NP	Y	Y	Y	B
Kwon et al [26]. (2022).	Y	Y	Y	NP	Y	Y	NP	Y	Y	Y	A
Ekfeldt et al [16]. (2015).	Y	Y	Y	Y	Y	N	N	Y	Y	Y	B
Rivers [20] (2016).	Y	Y	Y	Y	Y	NP	Y	Y	Y	Y	A
Finnegan et al [18] (2015).	Y	Y	Y	Y	Y	NP	N	Y	Y	Y	B

Note: ①Is there congruity between the stated philosophical perspective and the research methodology?②Is there congruity between the research methodology and the research question or objectives?③Is there congruity between the research methodology and the methods used to collect data?④Is there congruity between the research methodology and the representation and analysis of data?⑤Is there congruity between the research methodology and the interpretation of results?⑥Is there a statement locating the researcher culturally or theoretically?⑦Is the influence of the researcher on the research, and vice- versa, addressed?⑧Are participants, and their voices, adequately represented?⑨Is the research ethical according to current criteria or, for recent studies, and is there evidence of ethical approval by an appropriate body?⑩Do the conclusions drawn in the research report flow from the analysis, or interpretation, of the data?

Abbreviations: N, No; NP, not provided; Y, Yes

### Results of synthesis

This study uses the method of aggregative integration [12] to integrate the results, that is, to further organize and summarize the meaning of the collected results, so as to make the results more convincing, targeted and general. Researchers in understanding the various qualitative research philosophy and methodology of the premise, through repeated reading, analysis and interpretation of each research results, are summarized, integration, form a new category and form integrated results. Finally extracted the results of 17 studies [7, 9, 14–28], which were summarized into 10 new categories and formed 3 meta-themes. The categories are presented below with supporting subcategories and illustrative quotes from the original studies.

#### Theme 1: Mental state of military nurses during deployment

##### Feeling down

Military nurses are often frustrated by complex battlefield environments or natural disasters. For example, some nurses may be frustrated by the lack of equipment or supplies, or despair that they cannot save the lives of the wounded; They were frustrated that they could not do more for the wounded. Other nurses were depressed about life after witnessing the brutality of war.

*“I am afraid of the battlefield situation on the plateau, and do not understand the local dialect, I do not know how to carry out the rescue work, and I am worried that I have not done anything, dragging everyone down.”* [7].

*“You are going to be frustrated at the lack of resources”; “you are going to see young people slaughtered more or less and feel hopelessness at not being able to save their lives.’’* [14].

*“Nurses reported frustration at the time it took for patients to arrive, the extent of injuries, and that they could not do more to save some patients.”* [9].

##### Emotion management

During deployment, nurses use a variety of methods to vent their emotions and keep them positive. Such as, taking a shower, keeping a journal, talking to others, Mutual acceptance and respect. By adopting positive coping measures, they enable themselves to be competent in their caring role and increase their belief in caring.

*“After each surgery I went to take a shower, pouring out my heart in tears, washing myself changing to a clean uniform, then going back like a new person”* [28].

*“I’ve had some depression on and off since I came back from Vietnam. If I kept a journal maybe I could get a better handle on some of the things that happened to me over there”* [15].

*“Confide in you colleagues and don’t hold things in…I think that’s what kept us going real well”* [15].

##### Sense of responsibility

It is crucially important for a nurse to understand the mission, policies, and procedures of the armed forces and the part one is asked to play as a military nurse. They need to understand that the purpose of the military is to support, protect, and defend a country’s national security interests. Performing military missions will enable them to serve a greater purpose in life. As both soldiers as well as nurses, based on the sense of responsibility to make them in a state of crisis to protect and serve the people, which make them proud. Military nurses also have an inspiring role to play by example.

*“We worked together in the implementation of emergency rescue support tasks, filled with positive energy and a sense of honor, and strengthened our sense of mission”* [7].

*“To be something of a father-figure, to give the soldiers a feeling of safety. Keep your eye on your men so that they know they will be looked after if anything happens”* [14].

#### Theme 2: the experience of military nurses during deployment

##### The chaos

There are three main types of “chaos” here: Natural disasters and wars make the environment chaotic; the environment of disaster or war often makes the rescue work of nurses full of uncertainty, which leads to confusion in the team; chaos in the role of nurses during deployment.

*“You get over there, [combat] it [the chaos] becomes real, bullets are flying, we’re being mortared … all these injuries, people with broken bones, blown off arms, burns … [In disasters, initially] “It was pure chaos, triage was going on, treatment was going on, people [were] everywhere, lying on the conveyor belt, in wheelchairs, tons of elderly, some had no clothing, it was just a sea of people that you could not see through”* [22].

*“One of our biggest challenges in critical situations is ambiguity or confusion in roles. These programs help us to clarify different roles in critical situations”* [24].

##### Unique environment

This is different from the usual environment, its “Unique” is manifested as: the uncertainty of the war zone; patients with complex injuries, such as explosion injuries, penetrating injuries; lack of resources and poor health care; and the special natural environment at high-altitudes.

*“We did not know what to expect in a war zone”* [28].

*“I usually have the habit of taking a bath every day, the most difficult to adapt to the field toilet and bathing, bathing like a market, the toilet is very simple, what flying animals can appear, often the toilet has not yet waited, it is necessary to gather training”* [7].

*“The biggest headache for me was the sweltering heat of the tent during the day and the shivering cold at night”* [7].

##### Team support

Team support is important. Maintaining a cohesive team relationship can not only improve the efficiency of casualty rescue, but also provide psychological support to each other. During deployment, the team helps and supports each other, and they are like a family. In addition, successful teams need strong leadership to ensure that the task is completed smoothly.

*“We were working in harmony, with collaboration between us. In this way, we could overcome this difficult and stressful time”* [28].

*“The chief nurse knew her people. She knew the nurses. She had a feel for what was going on in the unit and she knew who and when she could pull them, and where the staff needed to be to get the job done to cut down on the confusion*” [9].

##### The need for specialized skills

Due to the special nature of war trauma, medical personnel lack knowledge and experience in its cause mechanism and operation principle. Other nurses noted their lack of experience in military nursing because they had not been deployed before. Therefore, according to the study, military nurses need to improve their professional skills before deployment.

*“I have not systematically received the training of the professional theoretical knowledge of war injury rescue, and I have a sense of panic about the lack of professional knowledge when facing the practical rescue”* [7].

#### Training needs for emergency care

##### Psychological training needs

Military nursing is different from traditional nursing in terms of military obligations and requirements. Firstly, nurses need to cultivate military values, responsibility, patriotism, and a sense of sacrifice. Second, in a battlefield or disaster environment, military nurses face a variety of scenarios, so it requires them to develop a positive mindset. Finally, they need to keep their confidence and overcome their fear.

*“I think professional education should begin with enforcement in mind, and it is necessary for nurses to cultivate a spirit of sacrifice and patriotism.”* [27].

*“Be secure in yourself and in your professional abilities and limitations. Be realistic in your expectations. You have to cope with the reality and deal with it, even though it is very, very hard”* [15].

##### Military training content needs

Nurses play an increasingly important role in military missions and are often deployed to different missions, such as humanitarian operations, natural disasters and public health emergencies. Therefore, it is necessary that they have the relevant knowledge, skills and abilities. And they suggest that it is best to train them in local customs and languages before deployment. The special nature of military medicine, they have a lot to learn in the military, including combat and trauma care areas; Chemical, biological, radiological or nuclear (CBRN) preparation/reaction, such as Combat Casualty Care Course, Emergency War Surgery Course, or Trauma Nursing Core Course, etc. In addition, in the plateau region, they also learn medical care under extreme conditions.

*“I think the emergency response capacity should be enforced, such as when we run into public health emergencies and natural disasters; s”* [27].

*“Now, I think we are dealing with these cultural aspects in all our operational readiness courses”* [15].

*“Fluid resuscitation on plains and plateaus is different; thus, we also need to learn medical care and nursing skills for extreme environments”* [27].

##### Training methods needs

Mixed training methods should be adopted in teaching. Among them, practice, scenario simulation and distance learning are effective training methods, for example, they participated in training exercises in a field training environment or simulation laboratory. At the same time, they should not forget that teamwork training is also important in training.

*“I think scenario simulation is a good way, because theory lectures are too boring and we need to put theory into practice”* [27].

*“When participating in professional education, trainees should take part in exercise to avoid only talking on paper”* [27].

*“We had teamwork training during that education program, and I was impressed with this activity, which provided training on team cohesion”* [27].

*“Tabletop exercises were unrealistic and less helpful. We did not practice for a mass casualty.”* [9].

## Discussion

This systematic review and comprehensive study discussed the experience and training needs of nurses in military hospital in altitude first aid. The findings of the review have shown that military nurses faced a lot of physical and emotional stress during deployment. These stressors came from lack of professional ability, inadequate professional preparation, chaotic battlefield environment and extreme natural environment and similar. Military nurses found reasonable ways to cope with stress in a variety of military Settings. They receive training to improve professional competence and self-efficacy, while external support from care managers and colleagues also plays a vital role. However, more strategies are needed to enhance this effect.

The comprehensive quality of the individual (including physical and psychological quality) has a crucial impact on the rescue mission of military nurses [8]. For rescue in various environments(aircraft carriers, hospital ships, evacuation aircraft, plateaus, hypoxia, cold, desert, Gobi, high humidity, low pressure, jungle, and other area), rescuers need to have good physical fitness, positive and optimistic psychological quality and self-adjustment ability, in order to maximize their own knowledge and skills of high quality play out [29]. However, the findings of this review [7, 9, 16, 17] indicate that military nurses may experience altitude sickness, fatigue, nausea, and even acute pulmonary edema when faced with a cold, oxygen-deprived altitude environment; faced with many casualties, they feel depressed, helpless, sad and even depressed. Therefore, military nurses should pay attention to physical training, enhance physical quality, to resist and adapt to extreme environment; nursing managers accurately their psychological state, timely guidance, tracking comfort. The findings of this review also suggest strengthening teamwork and support, which can help nurses support each other during periods of loneliness and provide quality care to wounded patients [6, 7, 22, 24]. Bonnie et al [30]. also suggests trying to change thinking and manage emotions by changing feelings and reframing experiences.

Knowledge and technology are the fundamental prerequisites for military nurses to accomplish rescue operations [31]. This review found that knowledge and skills were mentioned more frequently, indicating that knowledge and skills were the most concerned skills of nurses participating in deployment, and rich knowledge storage and skilled nursing skills are crucial to the first aid of the wounded. Other studies have also drawn a similar conclusion. For example, Harris [32] found that one unique aspect of clinical expertise in the context of military nursing is clinical diversity, and military nurses should not specialize in just one specialty, but should have multidisciplinary nursing knowledge and skills. Formulating a scientific and effective training program is helpful to improve the ability of military nurses. Caporiccio et al [33]. found continuing professional education (CPE) is widely recognized by nurses who learn the latest knowledge and skills through CPE, which has become the primary source for maintaining their competencies and ensuring better outcomes worldwide. The training including trauma care, combat knowledge, field nursing, the cultural customs and languages of the deployment place, chemical, biological, radiological or nuclear (CBRN) preparation/reaction(such as Combat Casualty Care Course, Emergency War Surgery Course, or Trauma Nursing Core Course, etc.) [8, 9, 14, 15, 27]. Learning barriers have family and work factors, trainees often did not want to attend training because they are worried about their children or heavy work, the learning environment is also an important factor, and the positive learning atmosphere organized by the staff can make the trainees full of passion for learning [34]. In addition, appropriate training methods have a positive effect on improving nurses’ professional skills. The main methods include practice, scene simulation and distance learning. And leaders should pay attention to teamwork training among medical staff [9, 27, 35]. Overall, making scientific training programs and creating a good learning atmosphere are helpful to improve the knowledge and technology of military nurses.

Competency is the key to affect the rescue mission of military nurses [31]. Competency is an important invisible feature for military nurses to complete rescue tasks, and is the driving force for other skills to play. Military nurses need to have the ability of organization and management, nursing risk prediction, nursing decision making, emergency handling and so on when performing rescue tasks [29, 36]. These are essential conditions for successful treatment. Some studies have shown that team members from different majors simulate operation and rescue tasks in non-task environments, which can effectively prevent the repetition of wrong behaviors by improving leadership, communication skills, teamwork, etc [24, 37]. Good communication and teamwork can also reduce the occurrence of adverse events during rescue [24]. Decisive decision-making ability becomes the key to winning survival time, and good emergency response ability can often avoid further damage [4, 29, 38]. Therefore, military nurses with good comprehensive ability can achieve the rescue effect of both efficiency and quality. Through simulation-based training, military nurses can improve their personal knowledge, skills, abilities, thinking and team ability [4]. Such as high-fidelity simulation could improve emergency management capabilities, team leadership, and basic nursing skills [39]; human patient simulators could improve their cognitive thinking and critical thinking skills [40]; hyper-realistic immersive training could improve the performance of multidisciplinary medical team members and facilitate effective collaboration between members and teams [41]. We found that military nurses are more willing to improve their ability through practice [27]. Consequently, it is suggested that the management should expand the practical training mode and combine various simulated training with simulated extreme environment to enhance the comprehensive ability and adaptability of military nurses to special environment.

### Strengths and limitations

The advantage of this study is that we not only searched medical databases but supplemented this with manual searches to ensure that studies were fully retrieved. Secondly, we conducted quality control, data extraction, and study quality assessment. Finally, the study is largely reflective of the dilemmas and needs of military nurse and is of great significance to military emergency care. However, there are some limitations to this study. Although the search strategy was thorough, some articles may have been missed, such as the gray literature. And the lack of detailed discussion on the potential influence of the researchers on some of the research studies suggests a possible bias of the findings of original studies.

## Conclusions

This qualitative systematic review reviews the experience of military nurses during deployment and analyzes the feelings, experiences, and needs of military nurses during military duty. In contrast, there is less research on emergency rescue operations in extreme environments such as high altitudes, which should be the focus of future exploratory research. Qualitative research in this area should address the lack of mental, physical, and professional preparedness of deployers by understanding the experiences of those with deployment experience in extreme environments. In the future, managers should design diversified, personalized training programs and training methods that are suitable for the deployment of military nurses in a variety of environments.

### Electronic supplementary material

Below is the link to the electronic supplementary material.

Supplementary Material 1

Supplementary Material 2

Supplementary Material 3

Supplementary Material 4

## Data Availability

Data used to support the findings of this study are available from the corresponding author upon request.
