# Supplementary material for: Experience and training needs of nurses in military hospital on emergency rescue at high altitude: a qualitative meta-synthesis
Source: BMC Nurs. 2024 Jun 3;23:370. doi: 10.1186/s12912-024-02029-1 (PMC11145869; doi:10.1186/s12912-024-02029-1)
Supplement: Supplementary file 1 — Supplementary Material 1 [file 12912_2024_2029_MOESM1_ESM.docx]

**Additional file 1: Search strategies**

Search strategies used for finding qualitative research articles about experience and training needs of nurses in military hospital on emergency rescue at high altitude. Number of retrieved articles is given in the right-hand column.

Date of Search: May 30 2023

**PubMed**

| S1 | nurse[Mesh, ti, ab] | 241,226 |
| --- | --- | --- |
| S2 | “military” OR “army” OR “air force” OR “navy” OR “warrior” [Mesh, ti, ab] | 365,059 |
| S3 | “emergency rescue” OR “disaster” OR “public health emergency” OR “rescue” OR “War readiness” OR “war” OR“experience” OR “education” OR “train”[Mesh, ti, ab] | 2,604,715 |
| S4 | “plateau” OR “highland” OR “high altitude” [Mesh, ti, ab] | 82,593 |
| S5 | “qualitative study” OR “interview” OR “focus group”[Mesh, ti, ab] | 267,855 |
| S6 | S1 AND S2 AND S3 AND S4 AND S5 | 341 |

**CINAHL**

| S1 | nurse[AB] | 328,070 |
| --- | --- | --- |
| S2 | “military” OR “army” OR “air force” OR “navy” OR “warrior” [AB] | 19,148 |
| S3 | “emergency rescue” OR “disaster” OR “public health emergency” OR “rescue” OR “War readiness” OR “war” OR“experience” OR “education” OR “train”[AB] | 488,324 |
| S4 | “plateau” OR “highland” OR “high altitude” [AB] | 6,420 |
| S5 | “qualitative study” OR “interview” OR “focus group”[AB] | 113,811 |
| S6 | S1 AND S2 AND S3 AND S4 AND S5 | 0 |
| S7 | S1 AND S2 AND S3 AND S5 | 46 |
| S8 | S1 AND S2 AND S4 AND S5 | 0 |
| S9 | S1AND S2 AND S5 | 82 |

**EMBASE**

| S1 | nurse[ ti, ab] | 181,514 |
| --- | --- | --- |
| S2 | “military” OR “army” OR “air force” OR “navy” OR “warrior” [ ti, ab] | 90,933 |
| S3 | “emergency rescue” OR “disaster” OR “public health emergency” OR “rescue” OR “War readiness” OR “war” OR“experience” OR “education” OR “train” [ ti, ab] | 187,674 |
| S4 | “plateau” OR “highland” OR “high altitude” [ ti, ab] | 81,432 |
| S5 | “qualitative study” OR “interview” OR “focus group”[ ti, ab] | 319,526 |
| S6 | S1 AND S2 AND S3 AND S4 AND S5 | 0 |
| S7 | S1 AND S2 AND S3 AND S5 | 10 |
| S8 | S1 AND S2 AND S4 AND S5 | 0 |
| S9 | S1 AND S2 AND S5 | 41 |

**PsycINFO**

| S1 | nurse[AB] | 70,167 |
| --- | --- | --- |
| S2 | “military” OR “army” OR “air force” OR “navy” OR “warrior” [AB] | 32,554 |
| S3 | “emergency rescue” OR “disaster” OR “public health emergency” OR “rescue” OR “War readiness” OR “war” OR“experience” OR “education” OR “train” [AB] | 727,137 |
| S4 | “plateau” OR “highland” OR “high altitude” [AB] | 2,632 |
| S5 | “qualitative study” OR “interview” OR “focus group”[AB] | 161,244 |
| S6 | S1 AND S2 AND S3 AND S4 AND S5 | 0 |
| S7 | S1 AND S2 AND S3 AND S5 | 16 |
| S8 | S1 AND S2 AND S4 AND S5 | 0 |
| S9 | S1 AND S2 AND S5 | 36 |

**Cochrane Library**

| S1 | nurse[ ti, ab] | 637 |
| --- | --- | --- |
| S2 | “military” OR “army” OR “air force” OR “navy” OR “warrior” [ ti, ab] | 33 |
| S3 | “emergency rescue” OR “disaster” OR “public health emergency” OR “rescue” OR “War readiness” OR “war” OR“experience” OR “education” OR “train” [ ti, ab] | 200 |
| S4 | “plateau” OR “highland” OR “high altitude”[ ti, ab] | 10 |
| S5 | “qualitative study” OR “interview” OR “focus group”[ ti, ab] | 126 |
| S6 | S1 AND S2 AND S3 AND S4 AND S5 | 0 |
| S7 | S1 AND S2 AND S3 AND S5 | 3 |
| S8 | S1 AND S2 AND S4 AND S5 | 0 |
| S9 | S1 AND S2 AND S5 | 34 |

**FMRS**

| S1 | nurse[TIAB] | 146,102 |
| --- | --- | --- |
| S2 | “military” OR “army” OR “air force” OR “navy” OR “warrior” [TIAB] | 287,083 |
| S3 | “emergency rescue” OR “disaster” OR “public health emergency” OR “rescue” OR “War readiness” OR “war” OR“experience” OR “education” OR “train” [TIAB] | 2,091,208 |
| S4 | “plateau” OR “highland” OR “high altitude”[TIAB] | 85,367 |
| S5 | “qualitative study” OR “interview” OR “focus group”[TIAB] | 322,026 |
| S6 | S1 AND S2 AND S3 AND S4 AND S5 | 331 |

**CNKI**

| S1 | SU=(‘军队护士’+‘文职护士’) | 208 |
| --- | --- | --- |
| S2 | SU=(‘灾害’+‘突发公共事件’+‘救援’+‘战备’+‘教育’+‘经验’+‘培训’) | 122 |
| S3 | SU=(‘高原’+‘高海拔’) | 1,337 |
| S4 | SU=(‘质性研究’) | 29,113 |
| S5 | S1 AND S2 AND S3 AND S4 | 8 |
| S6 | S1 AND S2 AND S4 | 12 |
| S7 | S1 AND S3 AND S4 | 13 |
| S8 | S1 AND S4 | 16 |

**VIP**

| S1 | M=(军队护士+文职护士) | 206 |
| --- | --- | --- |
| S2 | M=(灾害+突发公共事件+救援+战备+教育+经验+培训) | 74 |
| S3 | M=(‘高原’+‘高海拔’) | 1,456 |
| S4 | M=(质性研究) | 58,703 |
| S5 | S1 AND S2 AND S3 AND S4 | 0 |
| S6 | S1 AND S2 AND S4 | 9 |
| S7 | S1 AND S3 AND S4 | 0 |
| S8 | S1 AND S4 | 12 |

**Wanfang**

| S1 | (题名或关键词:(军队护士) or 题名或关键词:(文职护士) ) | 1,122 |
| --- | --- | --- |
| S2 | (题名或关键词:(灾害) or 题名或关键词:(突发公共事件) or 题名或关键词:(救援) or 题名或关键词:(战备) or题名或关键词:(教育）or题名或关键词:(培训）or题名或关键词:(经验）) | 5,750,827 |
| S3 | (题名或关键词:(高原) or 题名或关键词:(高海拔) ) | 126,658 |
| S4 | 题名或关键词:质性研究 | 397,824 |
| S5 | S1 AND S2 AND S3 AND S4 | 1 |
| S6 | S1 AND S2 AND S4 | 9 |
| S7 | S1 AND S3 AND S4 | 3 |
| S8 | S1 AND S4 | 17 |

**CBM**

| S1 | "军队护士"[常用字段] OR "文职护士"[常用字段] | 407 |
| --- | --- | --- |
| S2 | ("灾害"[常用字段] OR "Disasters"[常用字段] OR "灾害"[主题词]) OR "突发公共事件"[常用字段] OR "救援"[常用字段] OR "战备"[常用字段] OR ("教育"[常用字段] OR "Education"[常用字段] OR "教育活动"[常用字段] OR "教育"[主题词]) OR "培训"[常用字段] OR "经验"[常用字段] | 1,073,992 |
| S3 | "高原"[常用字段] OR ("高海拔"[常用字段] OR "Altitude"[常用字段] OR "高海拔"[主题词]) | 28,243 |
| S4 | "质性研究"[常用字段] | 8,210 |
| S5 | S1 AND S2 AND S3 AND S4 | 5 |
| S6 | S1 AND S2 AND S4 | 7 |
| S7 | S1 AND S3 AND S4 | 7 |
| S8 | S1 AND S4 | 11 |

TOTAL FOUND: 1070
