# Supplementary material for: Experience and training needs of nurses in military hospital on emergency rescue at high altitude: a qualitative meta-synthesis
Source: BMC Nurs. 2024 Jun 3;23:370. doi: 10.1186/s12912-024-02029-1 (PMC11145869; doi:10.1186/s12912-024-02029-1)
Supplement: Supplementary file 2 — Supplementary Material 2 [file 12912_2024_2029_MOESM2_ESM.docx]

**Additional file 3: Results of meta-synthesis**

| **Finding** | **Category** | **Synthesized findings** |
| --- | --- | --- |
| 1.The menacing environment of the battlefield (U) | Feeling down | Synthesized findings 1: Mental state of military nurses during deployment. |
| 3.The challenge of battlefield care(U) |  |  |
| 25.Nurse emotions related to the mass casualty experience(U) |  |  |
| 28.Preparing to deal with anxiety(C) |  |  |
| 63.Being Strong Versus Expressing Emotion(U) |  |  |
| 58.Changed self(C) |  |  |
| 77. Emotional suffering(U) |  |  |
| 78. Alone(U) |  |  |
| 79. Death, devastation, and loss(U) |  |  |
| 85.Negative changes(U) |  |  |
| 91. Enduring confusion(C) |  |  |
| 99.The plateau battlefield ambulance is under great psychological pressure and trouble(C) |  |  |
| 108.Ethical bearing(U) |  |  |
| 113. Fear of a New Circumstance(U) |  |  |
| 115.Nervous about Caring for Unfamiliar, Critically Ill Patients(C) |  |  |
| 117.Psychological Withdrawal(U) |  |  |
| 12.Advice about journaling(U) | Emotion management |  |
| 14. Advice about caring for yourself(C) |  |  |
| 15.Advice about support systems (U) |  |  |
| 16.Advice about talking about your experiences(U) |  |  |
| 28.Preparing to deal with anxiety(C) |  |  |
| 34.Learning to manage changes in the environment(C) |  |  |
| 64.Existential Growth(C) |  |  |
| 82. Team support(U) |  |  |
| 84. Positive changes(U) |  |  |
| 106.Perceived self-worth as a member of an interprofessional team(U) |  |  |
| 120.Showing Comradeship and Encouraging Each Other(U) |  |  |
| 135. Emotional/Mental Ventilation(C) |  |  |
| 6.The nurse to take responsibility(U) | Sense of responsibility |  |
| 29.Preparing by investigating one’s motives(C) |  |  |
| 37.Serving a greater purpose in life(U) |  |  |
| 54.Different work spirit(U) |  |  |
| 92. Devotion to duty (C) |  |  |
| 98.A strong sense of pride and mission in carrying out tasks(U) |  |  |
| 105.Valuing joint responsibility and collaboration (C) |  |  |
| 1.The menacing environment of the battlefield(U) | The Chaos | Synthesized findings 2: The experience of military nurses during deployment |
| 5.Field manners (U) |  |  |
| 19.Dealing with uncertainty about incoming casualties(C) |  |  |
| 32.Figuring out where I ‘Fit’ in all the chaos(C) |  |  |
| 61.Structured Versus Chaos(C) |  |  |
| 79. Death, devastation, and loss(U) |  |  |
| 83. The chaos is real(U) |  |  |
| 87.The structure is missing(U) |  |  |
| 132. Exposure to harsh scenes of war(U) |  |  |
| 4.The complexity of war trauma(C) | Unique environment |  |
| 5.Field manners(U) |  |  |
| 22.Ripple effects of a mass casualty event (C) |  |  |
| 45.A unique clinical environment (C) |  |  |
| 53.Unusual working conditions(U) |  |  |
| 60.Known Versus Unknown(U) |  |  |
| 66.Challenging place(U) |  |  |
| 131.Water supply and difficulties in hygiene maintenance(C) |  |  |
| 15.Advice about support systemst(U) | Team support |  |
| 4.On shifting from a providing care expert model to one empowering partnering relationship(U) |  |  |
| 18.Organizing for Mass Casualty Operations (C) |  |  |
| 26.Engaged participants by forming relationships(U) |  |  |
| 31.Preparing through insight into the unique meaning of the caring relationship(U) |  |  |
| 82. Team support(U) |  |  |
| 93. Establishing deep comradeship(C) |  |  |
| 105.Valuing joint responsibility and collaboration (C) |  |  |
| 112.Leadership with followership(U) |  |  |
| 120.Showing Comradeship and Encouraging Each Other(U) |  |  |
| 134. Maintaining cohesive staff relationships(C) |  |  |
| 4.The complexity of war trauma (C) | The need for specialized skills |  |
| 23.Enlarging the scope of nursing practice(C) |  |  |
| 26.Preparing for transition from civilian care(U) |  |  |
| 41.Defence Operational Nursing Competency (DONC)(C) |  |  |
| 42.Specialist training(C) |  |  |
| 100.Own professional knowledge of war wounded rescue is insufficient(C) |  |  |
| 101.Lack of survivability in the wilderness on the plateau battlefield(C) |  |  |
| 102.Ethnic minorities lack communication between their religious beliefs and common language(U) |  |  |
| 126. Military training content(C) |  |  |
| 130.War service without military experience(C) |  |  |
| 119. Heavy workload and low caseload (C) |  |  |
| 9.Conduct military training(U) | Psychological training needs | Synthesized findings 3: Training needs for emergency care |
| 17.Advice about lack of preparation(U) |  |  |
| 26.Preparing for transition from civilian care(U) |  |  |
| 80. Unprepared(U) |  |  |
| 121.Gaining Confidence(C) |  |  |
| 126. Military training content (C) |  |  |
| 127. Military medicine training content(U) |  |  |
| 9.Conduct military training(U) | Military training content needs |  |
| 13.Advice about training(U) |  |  |
| 17.Advice about lack of preparation (U) |  |  |
| 23.Enlarging the scope of nursing practice(U) |  |  |
| 30.Preparing by investigating one’s professionalism (C) |  |  |
| 39.Educational preparation(C) |  |  |
| 42.Specialist training(C) |  |  |
| 43.Individualised training requirement and consolidation of training(U) |  |  |
| 51.Professional development on operations(C) |  |  |
| 100.Own professional knowledge of war wounded rescue is insufficient(C) |  |  |
| 101.Lack of survivability in the wilderness on the plateau battlefield(C) |  |  |
| 118.Studying Hard to Provide Skilled Nursing(U) |  |  |
| 126. Military training content (C) |  |  |
| 138. Contributions to helping the wounded(C) |  |  |
| 18.Organizing for Mass Casualty Operations (C) | Training methods needs |  |
| 41.Defence Operational Nursing Competency (DONC) (C) |  |  |
| 43.Individualised training requirement and consolidation of training(U) |  |  |
| 50.AMSTC, MSV & HOSPEX(C) |  |  |
| 51.Professional development on operations(C) |  |  |
| 128. Training methods(C) |  |  |
