# Supplementary material for: Experience and training needs of nurses in military hospital on emergency rescue at high altitude: a qualitative meta-synthesis
Source: BMC Nurs. 2024 Jun 3;23:370. doi: 10.1186/s12912-024-02029-1 (PMC11145869; doi:10.1186/s12912-024-02029-1)
Supplement: Supplementary file 4 — Supplementary Material 4 [file 12912_2024_2029_MOESM4_ESM.docx]

**Additional file 2: Finding and illustrations**

**Lindblad, C. and B. Sjöström, Battlefield emergency care: a study of nurses' perspectives. Accid Emerg Nurs, 2005. 13(1): p. 29-35.**

| Finding | Illustration from study | Evidence |
| --- | --- | --- |
| 1.The menacing environment of the battlefield | There have been blasts when we were in Bosnia,detonations very close to us. . . you are exposed to risks that affect your efficiency and can influence your decision as to what to do.(p.32) | Unequivocal |
| 2.The unknown job demands | How little you can feel when. . . the first time you have to clamber out of the tank when the vehicle commander says Here we are then and while you hear the blasts around you, you have to climb out and treat someone. (p.32) | Credible |
| 3.The challenge of battlefield care | You have to be pretty tough to take the considerable strain;you are going to be frustrated at the lack of resources; you are going to see young people slaughtered more or less and feel hopelessness at not  being able to save their lives. (p.32) | Unequivocal |
| 4.The complexity of war trauma | BATLS is easy to follow once you get the hang of it, but it is still very, very difficult to apply in the field. It is designed for the emergency room where you have all your gadgets . . . you are just not going to have resources for the people with a really bad prognosis and then I will have to decide among them who to concentrate on. (p.32) | Credible |
| 5.Field manners | You have to be comfortable with weapons and vehicles, also the safety regulations connected.If you’re not used to these surroundings it can be very stressful. You can underestimate the dangers and expose yourself and your mates to an increased risk. The environment should be familiar so that you can focus on the injured . (p.32) | Unequivocal |
| 6.The nurse to take responsibility | To be something of a father-figure, to give the soldiers a feeling of safety. Keep your eye on your men so that they know they will be looked after if anything happens. (p.32) | Unequivocal |
| 7.Ability for decision-making | You cannot do any examination other than a quick visual inspection. Is the person dead or alive? Is he breathing? Is he conscious? What do his injuries look like? The decision not to start treatment requires a good deal more experience than making an attempt at treatment. (p.33) | Credible |
| 8.Experience from combat casualty | Basic military training here ought to be longer than the ordinary conscript period so that everyone concerned has at least been inside an APC, a CV 90 or tank and is familiar with that branch of weaponry. (p.33) | Credible |
| 9.Conduct military training | Reserve officer’s training is a good idea because it involves repeated periods of service so you do not forget everything you’ve learnt. I think the best way  is to get out there for some training, get used to the set-up, get your feet wet, feel the shakes, the pulse and stress that can come, you have to get into the context. (p.33) | Unequivocal |
| 10. Working experience | I am not afraid of telling people what to do . . . just the need for quick action and efficiency on the spot, knowing what to do instead of just standing there hesitating, you have it in you, there’s never enough time, you just have to get on with it, that’s a great help. . . . then there’s having seen an awful lot. You can cope with the psychological side of seeing trauma. (p.33) | Credible |
| 11.Practical application of research findings | There’s a lot to be learned from the railway crash in Norway a couple of years ago when ambulance men had to go around giving oral doses of Ketalar to folks who were trapped, that’s a picture to take with you.  You can land in a similar situation yoursel. (p.33) | Unequivocal |

**Scannell-Desch, E.A., Lessons learned and advice from Vietnam war nurses: a qualitative study. J Adv Nurs, 2005. 49(6): p. 600-7.**

| Finding | Illustration from study | Evidence |
| --- | --- | --- |
| 12.Advice about journaling | Be sure you start a journal because it will help you so much more when you come back. That’s the number one thing I would tell them.(P.603)  I’ve had some depression on and off since I came back from Vietnam. If I kept a journal maybe I could get a better handle on some of the things that happened to me over ther.(P.603) | Unequivocal |
| 13.Advice about training | Ever since that time, if I have anything to do with training, I try to make it as realistic as possible. Many times I’ll tell our folks, the only reason we exist in the peacetime arena is to learn, so we can transfer that learning to the wartime environmen. (P.603) | Unequivocal |
| 14. Advice about caring for yourself | I think I would advise them first of all to know themselves. To know what works best for them as far as coping mechanisms because it is a rough, stressful area. (P.604) | Credible |
| 15.Advice about support systems | People shouldn’t think they have to be super nurse, and that they should be all things to all people. A lot of it is interpersonal relations, getting along with other crew members because it’s such a team effort. I think during wartime more than any other time, whether you are a flight nurse or a ground-pounder, we all need each other .(P.604) | Unequivocal |
| 16.Advice about talking about your experiences | Try and encourage them to talk to one another. I think if you talk and get it out, you’ll find that the person sitting next to you feels the same way, and you could avoid a lot of your problems. (P.605) | Unequivocal |
| 17.Advice about lack of preparation | I was not prepared emotionally, physically, or professionally to deal with the kinds of patients we had to deal with in Vietnam.(P.605)  I wasn’t prepared for the environment. I don’t think any of us were.prepared for no windows, no doors, an open operating room, no running water, no electricity. It was certainly less than optimal, and it sure took some getting used to. (P.605) | Unequivocal |

**De Jong, M.J., et al., Mass casualty care in an expeditionary environment: developing local knowledge and expertise in context. J Trauma Nurs, 2010. 17(1): p. 45-58.**

| Finding | Illustration from study | Evidence |
| --- | --- | --- |
| 18.Organizing for Mass Casualty Operations | We conducted a nursing-run mass casualty. We owned it. I owned it with the head nurse in my ER [emergency room]. One of the things that the last unit taught us and that sort of clicked in us is don’t change procedure. What we learned was that the way we run the ER during high-flow patients should be the same way we run the hospital during a mass casualty. Same players, same responsibilities. Because our experience back home is, mass casualty, you bring in some surgeon to do triage who hasn’t done triage since the last mass casualty. We do triage  every day in the ER, the head nurse, because when four or five patients come in, that’s the first thing you do. So that was sort of passed on, not as concrete as that, but that was sort of passed on from the other group.(P.49) | Credible |
| 19.Dealing with uncertainty about incoming casualties | The first five vehicles showed up and not knowing what was coming, we sent some people inside to be fast-tracked. About the sixth or seventh vehicle was a five-ton truck, and the soldiers were all dead. I knew right then that we were in for it. So we lined the dead bodies up over on the side and we were grabbing sheets and covering them. Well, then it was just a flood. It was just an absolute flood. And, because we didn’t know how big it was, we didn’t have a real good system setup. People were just everywhere. We ended up with at least 60 casualties outside in the back, and then the rest had all been moved in. Plus, we were dropping choppers on the flight line. We were sending them out four at a time to Balad and to Baghdad. It was crazy. So there was actually a moment where I found myself standing in one spot spinning around. Not knowing where to go next. Because you didn’t know who you already helped. There was just a mass of people. (P.51) | Credible |
| 20.Developing systems to track patients | It was a one-way system, and you have to make sure that it stays a one-way system. Everybody came through the ER. Everyone except for those with very minor injuries, who wore the green triage tags, the walkie-talkie, who literally could help with the mass casualty and then be treated many hours down the road, came through the ER door, and everyone had a number. We didn’t go by names. Now, if you were an American it was your last four of your social security number. And if you were an Iraqi, we had a numbering system—for anyone who wasn’t an American, we had a numbering system. And what we would do is, we would assign someone at the door, and they would be—the triage took place actually before they hit the door, outside, on the sidewalk. So patients were tagged with a color before they came  through. We put yellows in one part of the ER that was manned by people who really didn’t work in the ER. Those were not as critical. They could be cared for by nurses from the ward or we pulled nurses from the CASF [Contingency Aeromedical Staging Facility], whenever we could, or just had the techs watch them. And honestly, the techs probably did as much as nurses. The reds were the most serious and  came directly through. Whoever was the chief trauma doctor we called “the trauma czar.” He was the person who made the order for who’s going to CT first. It was up to them to do the secondary triage and decide who was the most serious and who would hit the ORs first. (P.52) | Unequivocal |
| 21.Resource utilization | It was very frustrating, because I’m certified in critical care. I was the only one in the entire hospital to have a CCRN. They had Army technicians taking care of the ICU patients, but they put me in taking care of prisoners. Eventually, they brought me in because they had patients with ICP [intracranial pressure] monitors and people didn’t know how to work them. So, I went ahead and serviced the monitors, and they said okay, I guess we’d better put her in the ICU. (P.53) | Credible |
| 22.Ripple effects of a mass casualty event | So you see the mass casualty sort of roll. It hits the ER and the ER’s chaotic, and it hits the OR and the OR’s chaotic, but they’ve tapered down now, because everything’s shifted into the house, so the OR and ICUs pick up and then the wards start getting their piece. It sort of waves through the organization.  And, then on the other end, the wave sort of shifts out, to where we start sending these patients out [air transport].(P.54) | Credible |
| 23.Enlarging the scope of nursing practice | So many of us have seen so many of these things on a daily basis in mass casualty situations. We need somebody to intubate this guy! Why didn’t one of us think out of that box for a second? What stopped us from doing that next step? I don’t know. I could have probably gone in there and intubated him. I just had  my imaginary line there. I can’t cross that…. If you don’t have the resources, you’re going have to step outside your comfort zone, for sure. (P.55) | Credible |
| 24.Medical facilities under attack | It was very hard for people to manage situations like Fallujah, during Ramadan. We were getting mortared five times a day, and so the alarms were ringing all the time and then we started having mass casualties coming in from the Fallujah assault. I don’t know how many we had altogether, but several hundred—anyway—we’d hear the helicopters coming in. It was  just like MASH! (P.55) | Unequivocal |
| 25.Nurse emotions related to the mass casualty experience | I think the biggest thing for me was my feeling of helplessness.When we had a mass casualty from a huge explosion that devastated the Italian complex in Nasiriyah. The Italians didn’t have ORs and things like that that could be used to care for someone who had been injured to those extremes. So we got around 30 patients from them. I think the overwhelming response was that things were so well-organized, things flowed so well that my heart broke when we couldn’t repair all the problems.(P.56) | Unequivocal |

**Ekfeldt, B., R. Österberg, and M. Nyström, Preparing for care in a combat environment. International Journal of Caring Sciences, 2015. 8(1): p. 1-8.**

| Finding | Illustration from study | Evidence |
| --- | --- | --- |
| 26.Preparing for transition from civilian care | It is not easy not to prioritize the patient’s life as in civilian care. It is strange to think about having to return fire before you take care of an injured friend. But I am prepared to work like that if I need to, even if it feels strange. (P.4) | Unequivocal |
| 27.Preparing for work in a complex context | I thought about it in advance. How do I tell my colleagues how they should assist and what they should do? What should I report to the foreign helicopter that’s coming? How do I say this in English? I have to drill things like that. (P.4) | Credible |
| 28.Preparing to deal with anxiety | Sometimes it feels hard to be so far away. It can take eight hours by car for the nearest medically trained person to get there. So it is clear that everything is up to me if something happens.This can make you nervous sometimes. (P.4) | Credible |
| 29.Preparing by investigating one’s motives | The thing that could make me hesitate is that my son is 2.5 years old. You think about all the risks. But as my husband is an officer and has been away, he encouraged me to go.(P.5) | Credible |
| 30.Preparing by investigating one’s professionalism | Security in the professional role is crucial for the outcome in an environment like this. If I'm not safe with this I can’t perform under extraordinary operating conditions.(P.5) | Credible |
| 31.Preparing through insight into the unique meaning of the caring relationship | If anything happens to me, I hope my friends will be able to take care of me in the best way. We practiced nursing at home in training and with my buddies down in Afghanistan so I feel safe.(P.5) | Unequivocal |

**Elliott, B., Military nurses' experiences returning from war. J Adv Nurs, 2015. 71(5): p. 1066-75.**

| Finding | Illustration from study | Evidence |
| --- | --- | --- |
| 32.Figuring out where I ‘Fit’ in all the chaos | I would literally be standing on my front porch within 2 days of having seen some of the most horrifying trauma any human being could never imagine. And you’re trying to step into the role of  being a father, husband, neighbor, friend and you can’t. Your brain cannot handle that amount of change that quickly, especially after it has been under prolonged stress. . .That’s the hardest part, is one minute you’re overseas, the next minute you’re standing there with your kids. . . (P.1069) | Credible |
| 33.Feeling like it’s all so trivial now | I hated going to the stores because people would complain and I realize that my whole perspective on life just kind of changed, or beliefs that I had before kind of enhance. You get a global vision of things. You see what war’s like and you see what it does and  then you stand in line and hear somebody complaining about the wait. I would set stuff down and walk out, because I was so afraid I was going to turn around and say something really not too nice. (P.1070) | Credible |
| 34.Learning to manage changes in the environment | When I heard a pager, I just remember stepping back thinking... ‘I don’t have to respond to this...I’m home,’ kind of thing. (P.1070) | Credible |
| 35.Facing the reality of multiple losses | I think you have to get to know yourself all over again. I tried to reach out to people. I lost friends...Inside I just felt completely numb and empty, like I was looking out of a mask...I felt like just getting a bulldozer and getting rid of every last friend I have and starting over with people who didn’t know me before, because then I wouldn’t go through this.(P.1070) | Credible |
| 36.Working through the guilt and moving forward | I think I still felt guilty and also felt a little helpless and out of control being deployed when you’re not directly there and you can’t respond to situations... When I came home I just craved to gain control over those roles again because I felt the loss of control. (P.1071) | Unequivocal |
| 37.Serving a greater purpose in life | There’s so much terrible stuff that happened, but the amount of people that we saved. . .just knowing that I was a part of that. . .you can’t explain that to someone and explain how great you feel doing that and how the ultimate experience that the soldier that comes in is dead basically on your table and you bring them back to life, you know? (P.1071) | Unequivocal |
| 38.Looking at life through a new lens | It means the sacrifices that you see through all means. The sacrifices of the soldiers. The sacrifices of the caregivers who are doing that role day in and day out. Sometimes in the ER just literally ankle deep in body fluids and carnage and despair. Then you take care of the locals and you just can’t believe how horrible their lives are. And it’s just an eye-opening experience. For me it was a quite a learning experience. I think you grow through that.(P.1071) | Credible |

**Finnegan, A., et al., Preparing British Military nurses to deliver nursing care on deployment. An Afghanistan study. Nurse Educ Today, 2015. 35(1): p. 104-12.**

| Finding | Illustration from study | Evidence |
| --- | --- | --- |
| 39.Educational preparation | the defence nursing educational strategy provided military nurses with the appropriate academic requirement for deployment.(P.106) | Credible |
| 40.Newly qualified nurses | Newly qualified, I do think they need a substantial hospital piece into that role, and then consolidate their nurse training. It's like a driving test, you can legally drive; it doesn't mean you are a safe driver. (P.106) | Crediblel |
| 41.Defence Operational Nursing Competency (DONC) | I think the DONC in its conception is a sound piece; the problem is the delivery of the DONC. There is a standard that you expect people to be at, which perception differs from assessor to assessor, where realistically there has to be one standardised level of assessment.We don't have the method of delivering the competency for it. We almost take it partly on merit and that people have already undertaken Trust related courses. I think, between MDHUs that varies  because obviously PD (Practice Development) led and how the PD team feel they should be delivering. That is where the guidance is missing, and this need for a training package. It has standards that need to be there, but not the how to get to that point. (P.108) | Crediblel |
| 42.Specialist training | The only thing from an Army side is that they push their personnel quite quickly through. I want to do ITU, OK, you're on your course next month. Then they are in sudden shock mode, they come to us, and we try to get them a quick start and we do send the message back that this is a stressful place and you are disadvantaged against those already in the role.(P.109) | Crediblel |
| 43.Individualised training requirement and consolidation of training | You are given a whole host of qualifications to get through. For example, adult life support, paediatric life support. I do question the relevance of some of those. For me, it was a lot of information overload without being able to consolidate each section of learning before you are onto the next thing. Certainly things like APLS (Advanced Paediatric Life Support) are very stressful, you need a month lead up to that, and I did 3 courses of the nature within a 3-week period. As well as maintaining a daytime job. And trying to maintain a work life balance.(P.109) | Unequivocal |
| 44.Clinical preparation | it was felt that peacetime clinical preparation and placements was sufficient to prepare nurses for their operational role. However, for nurses completing clinical placements within SHC NHS Trusts, there were significant concerns.(P.109) | Crediblel |
| 45.A unique clinical environment | I think it is difficult because the casualties we get here are not the sort you see in the UK. You don't get 3 triple amps together etc.Our RTA (Road Traffic Accidents) are local nationals and they don't wear seat belts, they don't have proper cars. So we have different levels of types of patients…You certainly get trauma in MDHUs but not trauma to the extent that you have amputations and gunshot wounds and casualties with half a body missing. (P.109) | Crediblel |
| 46.Clinical placements and level 1 Trauma Centres | 4 years ago the throughput from HERRICK back in Birmingham was horrific. As a theatre nurse with nearly a quarter of a century of experience, my breath was taken away. I could not even compre-hend the level of injuries people were surviving and where do you even start preparing for that. Because the profile of RCDM and the number of military patients meant that there was a very strong military team there. I think that prepared us well.(P.109) | Crediblel |
| 47.Local military contracts and committed shifts | If I am on a committed shift, I'm trouble shooting in minor injuries,because I can make that flow through quicker. It's not often our guys will get in rhesus (Resuscitation) on a committed shift, because they  want to get their own guys in there, understandably. And they can use us more effectively elsewhere to take the pressure off a very busy department. (P.110) | Crediblel |
| 48.Rotation programmes | You get patients (On Deployments) with a much higher acuity than you might ordinarily see on a ward. So some high dependency nursing would be useful. Not necessarily ITU, but high dependency.  Because it is quite scary when they come around from ITU with all the lines and everything. They quite literally, we had a guy the other day who literally had been extubated as he was wheeled out of ITU into the ward. That is scary for a lot of guys. HDU (High Dependency Unit) experience without a doubt .(P.110) | Crediblel |
| 49.Paediatrics | Adult and paediatric piece, there is a distinction. And here, we see children with gunshot wounds, stabbing and also amputations.You need to get instance stability, cause (sic) you have a really low threshold because their circulating volume is so much less and they could have bleed out so much quicker. The time frame from point of wounding to getting here could be 20 minutes. Whether or not the initial triage has been done, they get here and they are just so much more unstable.(P.110) | Unequivocal |
| 50.AMSTC, MSV & HOSPEX | I just feel that realistic training is massively important. I don't think that MSV and MSA, because of the stresses that you are put under there, to achieve and get the ticks in the boxes, I don't think that it does address these issues. You are thrown, together, you are given all of these critical situations to deal with, and you are watching how you are behaving. People are putting you under pressure to take you out of your comfort zone, and you don't have time to address these things .(P.111) | Crediblel |
| 51.Professional development on operations | Despite all the advances in educational preparation, clinical placements and HOSPEX, there is still a requirement to develop technical and nursing skills once deployed.(P.111) | Crediblel |
| 52.Return to contingency and overseas placements | 7 or 8 years ago there was training in South Africa to acclimatise staff, in particular to poly trauma and ballistic trauma. Two people I know who did it found it very valuable. Again, people I know who have done surgical training in Denmark have found that very good.(P.111) | Crediblel |

**Rahimaghaee, F., et al., Nurses' perceptions of care during wartime: a qualitative study. Int Nurs Rev, 2016. 63(2): p. 218-25.**

| Finding | Illustration from study | Evidence |
| --- | --- | --- |
| 53.Unusual working conditions | Some of the wounded had hit landmines and were a wreck.I had to urgently help them. In that critical moment, gloves and masks and the like didn’t make any sense to us. With my bare hands, I put the wounded man’s intestine back inside his belly and sent him under operation with a large gauze. It was a waste of time to search for gloves and the like. (P.220) | Unequivocal |
| 54.Different work spirit | It was my own desire to go to the front line; my job was considered a sort of defense, a defense of the youth of my country. The youth were truly sacrificing their life. Under the influence of the soldiers’ self-sacrifice, I was also sacrificing myself. This spirit seemed to dominate everyone everywhere at that time. (P.220) | Unequivocal |
| 55.A real but informal classroom | We were receiving clinical training in practice during the war. I can confess that I learnt from the wounded more than I learnt at the university. We faced the wounded by ourselves during the war. . . the war cases and the care we provided during the war was the best opportunities for receiving clinical training mostly in the form of self-education.(P.221) | Unequivocal |
| 56.Professional self-achievements | At first, I didn’t like nursing, but during the wartime, the conditions, the responsibilities and the care I provided made me feel helpful, so I became actually interested in nursing. (P.222) | Unequivocal |
| 57.Outcomes for the professional community | Some indicators of professionalism are related to the  level of education, such as a master’s degree and a PhD; this is one indicator, and the other indicator refers to the professional organizations formed in nursing. Nursing is indirectly indebted to the war for these organizations. How did male nurses enter nursing? It was the consequence of the war. Institutions based on nursing as an identity (such as a nursing organization), academic nursing associations and professional nursing associations are mainly run by men who participated in the war. I’m sure that if there was no war, not as much men would enter nursing. (P.222) | Unequivocal |
| 58.Changed self | There are memories that still make me cry to this day and that have had a lasting effect on me. . .. A pregnant woman from the villages was taken to give birth, but was killed on the way by a blast wave or something else; both she and her baby. The physician asked us to deliver the baby; I begged them not to bother the poor woman for God’s sake, but we had to get the baby out of the mother’s womb. I was all crying; I still can’t forget that day. It’s really one of the bitterest memories of my entire life. It was very hard –very. (P.222) | Crediblel |

**Rivers, F.M., US Military Nurses: Serving Within the Chaos of Disaster. Nurs Clin North Am, 2016. 51(4): p. 613-623.**

| Finding | Illustration from study | Evidence |
| --- | --- | --- |
| 59.Nature of War Versus Nature of Disaster | When we were over there [war] it’s organized, you know exactly what [patients] you are getting.where you are moving them to.but over here [disaster] information is limited, disorganized, you didn’t know where you were going until you were rolling. (P.617) | Unequivocal |
| 60.Known Versus Unknown | [In war, we were given the training, so we knew what to expect . they told us .[what conditions were like] before we got there .what to expect.but indisasters, we knew very little before we got there. (P.618) | Unequivocal |
| 61.Structured Versus Chaos | [In the disaster] it was just like chaos. You are so used to structure and suddenly there is none .everything that you knew doesn’t exist anymore . that piece of the puzzle that’s normally organized before you get there doesn’t exist anymore. (P.618) | Crediblel |
| 62.Prepared Versus Making Do | [In the disaster].we used plastic covers [from our packing supplies] to make isolation rooms.for traction devices.we used filled 5-gallon water jugs and bottles of [povidone-iodine] Betadine. (P.618) | Crediblel |
| 63.Being Strong Versus Expressing Emotion | [Those of us who serve in the military] we are supposed to be the strong ones.but at the end . it becomes a bit more difficult [to keep it inside, it builds up] because you [just] can’t share everything that goes on there [in the disaster]. (P.618) | Unequivocal |
| 64.Existential Growth | I came away with a lot more . awareness of what goes on out there . what I experienced 3 years ago continues to stay with me . to improve how I . look  at other people and not to be judgmental . to take people for who they are and understand the situation where they are coming from.(P.619) | Crediblel |

**Tow, J.C. and D.B. Hudson, Lived Experience of the Warrior Nurse as an Advisor. Mil Med, 2016. 181(4): p. 328-33.**

| Finding | Illustration from study | Evidence |
| --- | --- | --- |
| 65.Challenging role | The warrior role requirements challenged the nurses as well.Although wearing of protective gear on a regular basis contributed to the acceptance of the warrior experience, it also madehaving a professional conversation more challenging. (P.330) | Credible |
| 66.Challenging place | Just the constant danger and threat of being a target of attack contributed to the challenge of this difficult place.(P.330) | Unequivocal |
| 67.Changed | To see that I’m resilient and that I can do something like that and make my children proud of me for doing that. (P.330) | Credible |
| 68.Be careful | Choosing strategies after careful consideration of  “social logical implications” while focusing on the specific position responsibilities was described as “really important” for this role. The ability to let those who were being advised to perform the tasks or a “hands-off” approach was described as an advisory relationship strategy that provided “their own success.” The collaborative teamwork atmosphere required judicious record keeping to assist the “next team coming in” to “learn from our lessons.” The role required a constant “planning ahead” while being mindful of security for self and teammates. (P.330) | Credible |
| 69.Be powerful | The role required strong inspirational behaviors to make changeswith potential global impact. (P.330) | Credible |
| 70.Be courageous | Courage was a required trait for warrior nurses because of the risky environment. Role expectations included the ability to lead with fear in a challenging place that often required working outside comfort zones.(P.331) | Unequivocal |
| 71.Be resourceful | With the lack of available information and  inability to prepare for the role, relying on and having confidence to use information from personal life experiences was an internal trait needed for the role. (P.331) | Credible |
| 72.Be impeccable | An almost impossible to attain expectation for warrior nurses was to be the example for other people. This expectation required an idealistic character portrayal at all times regardless of personal weaknesses or shortcomings. The high-pressure environment required constant portrayal of confidence as though role players were “on stage.” (P.331) | Credible |
| 73.Be malleable | Working with different cultural practices of nurses in Afghanistan required building an appreciation for their abilities and tolerance of differences. Warrior nurses were expected to flourish and shine brightly despite the unfamiliar location and lack of family presence as support. (P.331) | Credible |
| 74.Be caring | Caring about “the safety of our entire team” was the direct responsibility of the warrior nurses. Safeguarding the local population and caring enough to respect limited abilities and resources were external expectations. (P.331) | Credible |
| 75.Be resilient | Warrior nurses were expected to carry heavy equipment along with an enormous amount of responsibility on their shoulders. They were expected to handle the pressure while attempting to change current practices that have the potential to make global impacts. (P.331) | Credible |
| 76.Group essence | Connections made offered them a new under-standing and respect for humanity as “looking through the eyes of whoever it is that you are serving that creates the most success.” Warrior nurses and their families were proud of the many sacrifices made and “putting their lives on the line” to make a difference in a difficult environment that is “not for the light hearted.” (P.331) | Credible |

**Rivers, F. and S. Gordon, Military nurse deployments: Similarities, differences, and resulting issues. Nurs Outlook, 2017. 65(5s): p. S100-s108.**

| Finding | Illustration from study | Evidence |
| --- | --- | --- |
| 77. Emotional suffering | They called a MASCAL [mass casualty] and I went. There was a young male soldier; had to be in his early 20’s; I picked up his boots, ‘cause he just had a bloody boot, his foot was in it.You hear that and think, gosh that’s awful, but it’s nothing like seeing it. If a picture is worth a thousand words, then that is worth a million. (P.8) | Unequivocal |
| 78. Alone | I deployed as an N of one and sadly, I came back as an N of one, I was alone at home. (P.8) | Unequivocal |
| 79. Death, devastation, and loss | I was reflecting back toward the end, [just] how catastrophic a disaster is; how it affects the social economic stratus in a community, [from] the very rich to the poorest of the poor including the homeless. It affects businesses, human day to day existence and lives, and the entire community, folks who had lost everything other than themselves and their family. It was just so widespread and there was so much grief and sense of loss. (P.9) | Unequivocal |
| 80. Unprepared | I really did go into it [combat] very unprepared mentally. I didn’t know how unprepared I was until I got there, I was elbows deep and at that point it was too late to go back. (P.9) | Unequivocal |
| 81. Command support | There is the feeling of lack of support from my chain of command. I’ve been in the military 26 years. [During my combat deployment] this was the very first time in my career that I’ve felt like I was insignificant, didn’t count, and just left out, just forgotten about, that is a very hard thing to swallow.(P.10) | Credible |
| 82. Team support | The only thing you’ve got is one another. You learned to rely on each other. A lot of us keep in touch. You develop a kinship, you bond together; that bonding is a protective mechanism. We were all we had. It was a survival mechanism.(P.10) | Unequivocal |
| 83. The chaos is real | You get over there, [combat] it [the chaos] becomes real, bullets are flying, we’re being mortared … all these injuries, people with broken bones, blown off arms, burns. (P.11) | Unequivocal |
| 84. Positive changes | I was grateful for the experience. I wanted to be doing it [my job] so that probably made a huge impact on my perception of the experience although some parts were hard it was definitely survivable.(P.11) | Unequivocal |
| 85.Negative changes | Yes, I have seen terror in war. I have seen burned and tortured civilians who were barely alive;multiple amputations and assisted with keeping them alive. I have been in the ER [emergency room] full of screams of agony moments after two suicide bombers detonated bombs near our hospital. I have also seen the loss, prepared their bodies, putting them in body bags. It is a terror of war that I can't unsee; no matter how many years have passed. It is a terror of war.(P.12) | Unequivocal |
| 86.We didn’t Know | [In disasters] “there is level of uncertainty. You don’t know where the people are, you don’t know their names, their names are confused, you have an extra body and you don’t know where it came from.(P.13) | Unequivocal |
| 87.The structure is missing | [In combat] you are so use to structure and all of a sudden [in disasters] there is none, that piece is missing. Your mind just wanders back down to the desert [combat area] and it’s controlled.You have weapons but it [war environment] is very controlled, you have logistical support. But here [in disasters] everything that you knew that was right didn’t exist.(P.13) | Unequivocal |
| 88.Enemy | Even though there is overlapping [between disaster and war deployments] there is a big difference.There is a HUGE difference [in disaster deployments].I have been to war, but how do you confront an enemy that’s already passed through and is now causing havoc in another state when you are focusing on the people in the community? The [survivors] are not the  enemy.(P.14) | Credible |
| 89.Deployment tempo | Disaster is just so much more concentrated [than war]. In disaster, every waking minute you are doing something that has a beneficial effect to somebody and how quickly everything moves.In something like 92 hours, we air-evac’ed close to 2000 people. We loaded planes pretty much every 30 minutes to an hour, working around the clock.(P.14) | Credible |
| 90.Deployment departure | Because of the nature of who we are [as military], there is a focus on being prepared, your bags are packed for combat, ready to go to war. You know you are going to war months out, the notice is given, but disaster deployment is much quicker, no time for planning, just go.You don’t have much time to really plan for a disaster deployment, you must get there quickly.(P.15) | Credible |

**Han, J.J., The Lived Experience of Korean Female Military Nursing Officers During the Vietnam War. J Transcult Nurs, 2019. 30(5): p. 471-477.**

| Finding | Illustration from study | Evidence |
| --- | --- | --- |
| 91. Enduring confusion | After nine days on the ship, I finally could see the land, far away.How beautiful it might be! The sea breeze blowing in my face was as soft as a silk scarf. But in the middle of that night, suddenly it became as bright as day due to the bursting of star shells. Everyone shouted and jumped out of the tent. From then on, we wore helmets even during sleep to protect us from brain injuries and to survive.(P.3) | Credible |
| 92. Devotion to duty | Even when I was going to the stream to wash my face, I was afraid that the Vietcong would be hiding in the trees and would shoot me. Whenever I felt fear, I told myself not to worry about anything other than thinking about how best to do my work on the battlefield. As an officer, I thought it would be an honor if I died there.(P.3) | Credible |
| 93. Establishing deep comradeship | If there was an engagement, helicopters evacuated the casualties first, and then, after a while, the corpses were flown in. There was no decent morgue at the time, so we put up a tent on the other side of the operating room and laid the bodies there temporarily. The corpses increased in number but no one wanted to go in the tent due to fear. I wanted them to be clean on their way to the heaven, so I went to the morgue and cleaned the blood off the bodies as much as possible. I was upset and angry because I couldn’t do anything about the young Korean soldiers’ deaths. (P.4) | Credible |
| 94.Realizing the dark side of war | A local female aid at our hospital said that she had cooked for the Vietcong the previous night. I told her not to do that for the enemy forces. She said the Vietcong was her cousin. I realized that they had no desperate desire to win this war and that this was not the same situation as the Korean War, when the South and North were fighting fiercely. (P.4) | Credible |
| 95.Being discriminated against as females | In contrast to the other department head officers, the chief nursing officer was not assigned a private military jeep. I explained to the troop commander the need for it to supervise the night shift nurses, who worked in the hospital, a long way from my quarters. Soon, he supplied a jeep for me to the hospital. However, the logistics officer kept it from me and tried to give it to another male officer. (P.4) | Unequivocal |
| 96. Achieving and being rewarded | I was the first nursing officer to complete an advanced Vietnamese language course and to work as a member of the civil affairs and psychological warfare group. Of course, working in the group always carried danger, but as an officer, I thought I should dedicate myself to the mission. In the end, I received the Order of National Security Merit from our president. (P.4) | Unequivocal |
| 97.Growing as leaders | When I returned from the Vietnam War, I always had challenges while working at the military hospital. I think I got the courage to be able to do anything when I came back from that difficult experience in Vietnam, as well as the confidence and the drive to take the bull by the horns instead of dodging problems. (P.5) | Credible |

**Xing, L. and X. Feng, A qualitative study of army civilian nurses’ experience on battlefield rescue in the northwest plateau theater. Journal of Nursing Administration, 2020. 20(03): p. 184-188**

| Finding | Illustration from study | Evidence |
| --- | --- | --- |
| 98.A strong sense of pride and mission in carrying out tasks | The implementation of the support task embodies a good team spirit, is not afraid of hardship or tiredness, deeply feels the importance of war wounded ambulance, and feels extremely proud. (p.185) | Unequivocal |
| 99.The plateau battlefield ambulance is under great psychological pressure and trouble | Although it is really inconvenient for female comrades to participate in the hospital training mission, especially during the menstrual period, they do not receive special care during the training. (p185) | Credible |
| 100.Own professional knowledge of war wounded rescue is insufficient | I feel that the skills of war wounded ambulance are very practical, and there are too few opportunities for this kind of theory and operation in school. (p.185) | Credible |
| 101.Lack of survivability in the wilderness on the plateau battlefield | I usually have the habit of bathing every day, the most difficult to adapt to the wild toilet and bathing, bathing is like rushing to the market, the toilet is very simple, any flying animal can appear, often the toilet has not arrived, to gather for training. p.185) | Credible |
| 102.Ethnic minorities lack communication between their religious beliefs and common language | During the disaster relief in Yushu, some of the wounded in Tibetan areas usually gave white hada in order to express their gratitude, but my hands were full of dirty mud, and I politely declined for fear of getting dirty, causing the wounded in Tibetan areas to misunderstand that I did not know how to be polite...... It is suggested that the training of hospitals can increase the ethnic and religious beliefs related to different ethnic minorities in the plateau area, so as to enhance the trust between nurses and patients. (p186) | Unequivocal |

**Vafadar, Z., M.H. Aghaei, and A. Ebadi, Military nurses' Experiences of Interprofessional education in Crisis Management: a Qualitative Content Analysis. J Adv Med Educ Prof, 2021. 9(2): p. 85-93.**

| Finding | Illustration from study | Evidence |
| --- | --- | --- |
| 103.Professional mutual recognition | It was an occasion for knowing each other. Unfortunately, in daily workflow, there isn’t any opportunity for us to interact and have mutual understanding, and when a crisis suddenly arises, we have to solve a problem together without having mutual understanding and shared knowledge and this is so dangerous….. Oh... problems start here. (p.89) | Unequivocal |
| 104.Shared mental models | Despite the different opinions and knowledge of each of us with respect to our professional role, in order to solve the problem in the presented scenarios, we had to share our views and come up with an agreed conclusion. (p.89) | Unequivocal |
| 105.Valuing joint responsibility and collaboration | I have participated in many maneuvers, but in them, the interaction between professions was not seen at all…, but at this maneuver, it was very amazing for me that people of different professions sit together and exchange their ideas and information. In previous maneuvers, each one was just…, we focused on our work, not on relationships and collaborations that could strengthen and move the work forward. (p.89) | Credible |
| 106.Perceived self-worth as a member of an interprofessional team | My good experience in the group was mutual acceptance and respect so that I could easily express my views without worrying about ridicule or being judged. It was a wonderful experience for me. I felt proud to be a member of an interprofessional team and to have the opportunity to show my abilities to others and my abilities were interesting to them. (p.90) | Unequivocal |

**Varpio, L., et al., Delivering patient care during large-scale emergency situations: Lessons from military care providers. PLoS One, 2021. 16(3): p. e0248286.**

| Finding | Illustration from study | Evidence |
| --- | --- | --- |
| 107.Mission focus | When MIHTs engage in patient care, there is an overarching objective—a mission—that informs and directs the team’s efforts.As one participant explained: “it’s important that all the teams have an understanding that the overall command mission is to support war-fighter readiness and taking care of their families. Everything we do is to support that.” (P.5) | Unequivocal |
| 108.Ethical bearing | MIHTs often confront moral dilemmas when delivering patient care, especially in large-scale emergency responses. The entire MIHT agreed that “it was the right thing to try and save their [enemy combatant’s] life” as well as the soldier’s life, despite the fact that surgical resources were in limited supply. MIHTs regularly face such dilemmas and need to embody clear ethics and moral strength. (P.6) | Unequivocal |
| 109.Situational awareness | A MIHT member must have his/her “head on a swivel” to stay safe. When MIHTs offer care, team members must continually read the environment to understand the physical situation and the resources available therein, so that they can adjust their actions straightaway. (P.6) | Credible |
| 110.Adaptability | In his normal work as a general surgeon, he would not be called up on to care for a patient with a broken leg because an orthopedic surgeon would be brought into the team. But, as a MIHT collaborator working in contexts where no other surgeon is available, he needs to be ready to provide this care. As he explained, MIHTs need versatile clinicians: “you have to have the high caliber clinician who also has adaptability outside of their specific trained area.” Adaptability requires MIHT collaborators to be physically and mentally capable; it necessitates being flexible, resourceful, and clinically prepared to provide patient care in sub-optimal environments. (P.8) | Unequivocal |
| 111.Unencumbered hands | At a moment’s notice, [to] be able to manage multiple complex trauma patients at the point of injury while there’s still shooting and blowing up going on. There is no time to be hampered by policies stating that a medic is not qualified to clamp an artery. If the medic has the skill and if the skill is needed, the medic will be asked to use that skill. (P.8) | Unequivocal |
| 112.Leadership with followership | Because of your role, you [the physician] end up a lot of times being the team leader, but part of being a leader is also being a follower and being smart enough to know that the nurse knows what he’s doing.” As situations dynamically evolve, all MIHT members are prepared to be both leaders and followers. (P.9) | Unequivocal |

**Kwon, Y.H., H.J. Han, and E. Park, Nursing Experience of New Nurses Caring for COVID-19 Patients in Military Hospitals: A Qualitative Study. Healthcare (Basel), 2022. 10(4).**

| Finding | Illustration from study | Evidence |
| --- | --- | --- |
| 113. Fear of a New Circumstance | I thought it may be too much for new nurses to care for COVID-19 patients while wearing a powered air-purifying respirator (PAPR) without being familiar with basic skills. I was worried that I may be infected while caring for COVID-19 patients and that I may transmit the infection to my family and others around me. (P.6) | Unequivocal |
| 114.Communication Difficulties with Isolated Patients | An elderly patient felt frustrated about the negative pressure facility itself. No matter how many times I explained, the patient tried to open the automatic door with force and broke the doors. Some family members asked me to deliver food to the patients. It was difficult. (P.6) | Unequivocal |
| 115.Nervous about Caring for Unfamiliar, Critically Ill Patients | The patients were not capable of independently caring for themselves. So, we had to do everything, from basic personal hygiene management to L-tube feeding, cleaning urine and feces, and changing their position in bed. (P.6) | Credible |
| 116.Physically Exhausted | The gloves and protective suit really gave me a lot of difficulties as a new nurse. Fatigue and exhaustion were severe. The work was vigorous, and I became tired and more sensitive. (P.6) | Unequivocal |
| 117.Psychological Withdrawal | When I thought I really could not do the given task, I sometimes asked a senior nurse outside (the isolation room). I felt ashamed and sorry that I could not even see their faces. (In the isolation room) I experienced great pressure as I had to perform the tasks alone. I wanted to do everything perfectly, the way the patients wanted. I was disappointed that I was not able to do so, and I lost confidence. (P.7) | Unequivocal |
| 118.Studying Hard to Provide Skilled Nursing | I watched videos on core skills, reviewed the skills on my own, and went to work. Changing the position of the patients on the bed is supposedly a simple task. However, I have only tried the task using a model in school. I had no practical experience and had to review the method on my own. I realized that studying from a book and actually caring for patients as a nurse were completely different. (P.7) | Unequivocal |
| 119.Searching for Own Know-How | I think it’s helpful to go into the isolation room and simulate in your head which room to enter and which skill to perform. In a general ward, if you did not bring a tourniquet for a lab test, you can simply go to the station and bring a tourniquet. But in isolation rooms, you have to tell colleagues outside the room to deliver the items. This takes twice the time and gives additional tasks to the colleagues outside the room. So I think it is important to minimize such things. (P.8) | Credible |
| 120.Showing Comradeship and Encouraging Each Other | I think the most difficult time was when I felt lonely. But there were a lot of people around me who I could understand and empathize with. I felt relieved when I talked to them on the phone.We were of different ranks. But they were my senior and junior colleagues. We shared sorrows and joys, and our bonds became much stronger. (P.8) | Unequivocal |
| 121.Gaining Confidence | Caring for COVID-19 patients is different from working in the general wards. (omitted). When I see myself solving problems without major difficulties or issues during busy times, I feel that I have adapted to the work. (P.8) | Credible |
| 122.Striving for Patient-Centered Care | While I was taking care of a patient, the patient’s condition deteriorated, and the patient was transferred to a higher-level hospital. Maybe I grew as a nurse. The experience was heartbreaking and difficult. I was scared. I was sorry to see the patient sick and being transferred. I felt a sense of duty to do better. (P.9) | Credible |
| 123.Thinking Critically | I think that the experience of working with experienced medical personnel to nurse COVID-19 patients has helped to improve the level of care and nursing capacity of military hospitals. Despite the lack of labor and requests for support, we never received quicker feedback or political, physical, and financial support. They only ‘thanked’ us in words, which felt foreign. (P.9) | Unequivocal |
| 124.Feeling Proud as a Military Nursing Officer | I felt proud, rewarded, and happy hearing messages like ‘thanks to you I recovered speedily.’ Looking back at it later, I think it will be a very happy moment in my life. (P.9) | Unequivocal |

**Ma, H., et al., Continuing professional education experiences and expectations of nurses in Chinese military hospitals: A quantitative and qualitative study. Nurse Educ Today, 2023. 120: p. 105645.**

| Finding | Illustration from study | Evidence |
| --- | --- | --- |
| 125. Military missions | We are taking turns to be deployed for humanitarian operations. It would be better to train us in the local customs and language before deployment; thus, we could have a basic understanding. This training program should be offered regularly, not only after the deployment notification. (p.4) | Credible |
| 126. Military training content | I think professional education should begin with enforcement in mind, and it is necessary for nurses to cultivate a spirit of sacrifice and patriotism. Military training should also be paid attention to. As military personnel, we shoulder many responsibilities. We need to increase military training and practice our nursing skills to prepare for future missions. (p.4) | Credible |
| 127. Military medicine training content | I think besides psychological nursing and contagious disease management, trauma and combat care are also important in field care. Nurses with less work experience can learn basic trauma care. Advanced nurses need to learn to cooperate with doctors to do damage control surgery or to provide advanced life support.(p.4) | Unequivocal |
| 128. Training methods | I think scenario simulation is a good way, because theory lectures are too boring and we need to put theory into practice. Distance learning is also a good method to consider when learning theoretical knowledge, besides training programs in university and college. (p.4) | Credible |
| 129.Professional development paths | I think management is an important part of professional education, such as management thinking and management tools. In addition to management, research and specialist nursing, such as emergency care and critical care, are also essential.(p.5) | Credible |

**Segev, R., Learning from critical care nurses' wartime experiences and their long-term impacts. Nurs Crit Care, 2023. 28(2): p. 253-260.**

| Finding | Illustration from study | Evidence |
| --- | --- | --- |
| 130.War service without military experience | We did not know what to expect in a war zone. We had no knowledge about either using our weapons or how to manage a field hospital. We did it in real-time, using our common sense, using our skills from civilian wards.(p.256) | Credible |
| 131.Water supply and difficulties in hygiene maintenance | From the beginning of the war we had not taken a shower. We only washed our faces, hands, and genitals. We had to face an insufficient water supply. At one time we would have water, and later we would not. It was a serious problem to maintain the hygiene of our hands and the medical equipment. (p.256) | Credible |
| 132. Exposure to harsh scenes of war | Exposure to war's horror was the most significant issue the nurses related. “I remember the clotted blood with its uniquely acidic smell. Seriously wounded soldiers came to us with their chests and abdomens open. They showed up dirty with soil, blood, and even the food they had for lunch spread all over their open chests”. For another, the interview elicited this memory: “The sight of the burned soldiers, the sounds of helicopters, and the pounding of nails to make coffins for dead, refuse to leave me until today”. (p.256) | Unequivocal |
| 133. Improvisation | Nurses used improvisation and creativity to overcome the medical demands they faced by providing critical care in battlefield conditions. “We faced a lack of medical equipment, so we called our friends overseas and they sent us a lot of necessary items. If anyone from the hospital went home on leave, we asked them to bring back specific things”. One nurse described needing an orthopaedic nail to repair an injured soldier's elbow: “I sent a soldier outside the operating room to sterilize a non-medical nail, under fire. Unfortunately, the soldier whose elbow we fixed did not survive”. (p.256) | Unequivocal |
| 134. Maintaining cohesive staff relationships | We were working in harmony, with collaboration between us. We stayed in tents together—male and female. Our commanders ordered us to separate the tents by gender, but we refused to do so. In this way, we could overcome this difficult and stressful time. (p.257) | Credible |
| 135. Emotional/Mental Ventilation | After each surgery I went to take a shower, pouring out my heart in tears, washing myself changing to a clean uniform, then going back like a new person. For another, the meetings between several field hospital staff members to exchange equipment and blood products enabled us to ventilate and share the emotional burden. That helped us to move on with renewed energy. (p.257) | Credible |
| 136. Avoidance and Denial | After a resuscitation event, I went outside and did not want to meet anyone. We worked like robots and did not talk about the war. We also avoided learning the soldiers' names. We were afraid to encounter someone we knew. (p.257) | Credible |
| 137. Organizational and management aspects | We managed the human resources throughout the hospital. Our civilian experience enabled us to act by prioritizing according to the urgency of missions. We found ourselves taking intimate care of all the women in the military base zone. We also took care of the dignity and memory of those who died by collecting their personal belongings and later giving them to their families. Another added: we were always thinking about the hospital's needs. We maintained the medical equipment and prevented the waste of materials for dressing wounds. We worked after our shift ended and gave our turn to go for a short vacation to those who had families and children. (p.257) | Unequivocal |
| 138. Contributions to helping the wounded | Most participants acknowledged their contributions to helping injured soldiers. Many reported that reactions from the wounded gave them energy and justified their service in a hostile warzone environment. One recounted: “Soldiers who felt the nurse's hand or even her feminine voice gained strength and hope to fight for their lives. They told us this”. (p.257) | Credible |
| 139.Expectations from military and governmental authorities for acknowledgement and recognition | Some of us got a certificate of appreciation by mail. We did not get the deserved attention for our contribution. At that time, we did not think our story should be publicized, because we did not perceive it as a special act. (p.257) | Credible |
